# Medical News

**Published:** 1854-11

**Authors:** 


### THE MEDICAL EXAMINER.

PHILADELPHIA, NOVEMBER, 1854.

### MEDICAL NEWS.

The deaths from Cholera in London, during the week ending Sept. 16, were 1549. Upon this diminished mortality, the London Lancet of Sept. 23 observes: "We are thus glad to find that the deaths from cholera, instead of 2050 are 1549, or 501 less than the deaths from the same cause in the preceding week. The total deaths in the present eruption have been 7669; in the eruption of 1849 the deaths by cholera up to the same date, within one day, were 11,825. In both of the eruptions the mortality was highest on nearly the same day of September; its decline commenced in the corresponding week; and we may now sanguinely hope that it will descend as rapidly as it did in the autumn of 1849. But no exertion should be spared to save the thousands whose lives are still threatened; and the dread lesson, before regarded so little, should never be forgotten,—that men can no longer drink polluted water—breathe impure air—neglect sanatory measures year after year—with impunity." The mortality in the following week was 1284, and in the week ending Sept. 30th, 754. The total mortality to Sept. 30th, 1854, has been 9707. In 1849 there were 13,098 deaths up to Sept. 29th. It should be remembered, however, that the present epidemic began much later in London than it did in 1849.

Special meetings have been held by the Physicians and Surgeons of the New York Hospital, by the Medical Faculty of the University of the City of New York, and by the members of the New York Medical and Surgical Society, to express their sense of the deep loss sustained by the profession in the death of Dr. Swett. Dr. John Watson has been requested to prepare a memoir of his life and services, to be delivered at the New York Hospital, of which Institution Dr. Swett was one of the Physicians.

Died, on the 20th of September, in New Orleans, of yellow fever, Valentine Mott, Jr., M.D., son of Prof. Mott, in the 33d year of his age, on his way to New York.

Dr. Carter P. Johnson, Professor of Anatomy in the Medical College of Virginia, was, we deeply regret to see, one of the passengers on board of the ill-fated Arctic.

Among the victims of the yellow fever at Savannah, we notice the names of Drs. P. H. Wildman, F. W. Schley, S. N. Harris, T. M. Ellis, and C. H. Welles.

In a note appended to Dr. Mason's "Case of Rupture of the Uterus, in which the operation of Gastrotomy was successfully performed by John Neill, M.D.," published in our last number, we stated our belief that it was the first instance of recovery in the United States. In this we were mistaken. Dr. John T. Gilman, of Portland, Maine, reported a case in the April No., 1854, of the American Journal of Medical Sciences, in which he operated successfully, twenty-one hours after the uterus had been ruptured. We owe an apology to Dr. Gilman for our inadvertent omission of his report, and make the only reparation in our power by this acknowledgment. We record his article in the present number of the Examiner.

